# Dysentery as the only presentation of COVID-19 in a child: a case report

**DOI:** 10.1186/s13256-021-02672-1

**Published:** 2021-02-08

**Authors:** Marjan Tariverdi, Nazanin Farahbakhsh, Hamed Gouklani, Fatemeh Khosravifar, Mohammad Tamaddondar

**Affiliations:** 1 Department of Pediatric, Clinical Research Development Center of Children Hospital, Hormozgan University of Medical Sciences, Bandar Abbas, Iran; 2 Department of Pediatric Pulmonology, Mofid Children’s Hospital, Shahid Beheshti University of Medical Sciences, Tehran, Iran; 3 Infectious and Tropical Diseases Research Center, Hormozgan Health Institute, Hormozgan University of Medical Sciences, Bandar Abbas, Iran; 4 Student Research Committee, Hormozgan University of Medical Sciences, Bandar Abbas, Iran; 5 Department of Nephrology and Internal Medicine, Shahid Mohammadi Hospital, Hormozgan University of Medical Sciences, Bandar Abbas, Iran

**Keywords:** COVID-19, Dysentery, SARS-CoV-2, Pediatric, Case report

## Abstract

**Introduction:**

The coronavirus disease 2019 (COVID-19) pandemic has caused irreparable damage to society, and the damage continues. Pediatricians are confronted with COVID-19 in a variety of presentations, which may lead to delayed diagnosis and treatment. Early diagnosis of the disease plays an important role in preventing transmission of the virus in the community.

**Case presentation:**

Here we report a 27-month-old previously healthy Iranian female child who presented with fever and bloody diarrhea, diagnosed with COVID-19 based on contact history, exclusion of enteric bacterial pathogens and parasites, and positive stool and nasopharyngeal severe acute respiratory syndrome coronavirus 2 (SARS-CoV-2) reverse transcriptase polymerase chain reaction (RT-PCR) tests. The patient had viral shedding for more than a month.

**Conclusions:**

The pediatric population usually does not present with typical clinical features of COVID-19, which are respiratory involvement. Dysentery may be the only presentation of this disease, and long-term isolation should be considered, as the viral shedding may last for more than a month.

## Introduction

In December 2019, an epidemic of what would later become known as coronavirus disease 2019 (COVID-19), caused by severe acute respiratory syndrome coronavirus 2 (SARS-CoV-2), began with the report of a pneumonia with an unknown cause from Wuhan, Hubei Province, China. The pathogen was identified as a novel coronavirus in early January, and finally on January 30, 2020, the World Health Organization (WHO) declared the novel coronavirus a public health emergency of international concern [1].

As COVID-19 is a novel disease, there is still a large information gap in its clinical presentations in the pediatric population. Consequently, reporting every new manifestation of this disease can help physicians in timely diagnosis and treatment of similar cases. Despite the preliminary data on the presentation of COVID-19, which was focused on respiratory symptoms, as the pandemic persists and more cases are diagnosed, other clinical features of COVID-19 have been identified [1]. Gastrointestinal (GI) symptoms of COVID-19 including diarrhea, nausea, vomiting, abdominal pain, and GI bleeding have been mentioned in previous studies [2–4], but to date no dysentery with COVID-19 in the pediatric population has been reported. We aimed to describe a 27-month-old female with COVID-19 who presented with dysentery as the only presentation of this disease.

## Case presentation

A 27-month-old Iranian female child was brought to our center due to fever, vomiting, and loose stool for 2 days prior to admission. The patient had been seen in an outpatient clinic, but as conservative care was not effective, she was admitted to the infectious ward. She had no significant past medical history or any comorbid medical conditions. There was no recent travel history, and no members of her family had the same symptoms except for the grandfather, who had experienced fever and malaise for a week. On admission, the patient’s temperature was 39 °C, and she had sunken eyes, dry mucosa, and fair skin turgor. Laboratory data results were as follows: stool exam, 10 white blood cells (WBCs)/high-power field (HPF); 10 red blood cells (RBCs)/HPF; many pus cells. Stool culture was negative for enteric bacterial pathogens and parasites. Other laboratory results included C-reactive protein (CRP) 29 mg/l, complete blood count (CBC); WBC 3400/µl, 27% lymphocyte and 52% neutrophil. According to the guideline of this center, intravenous hydration and ceftriaxone were administered as approved empirical therapy in children with dysentery. No appropriate clinical or laboratory response was observed. On the second day of admission, the patient developed abdominal cramps following bloody diarrhea and an increase in CRP level to 69 mg/l. Meanwhile, we were informed that the grandfather had subsequently tested positive for SARS-CoV-2 by reverse transcriptase polymerase chain reaction (RT-PCR) test, and the patient had close contact with him. We ordered a chest X-ray (CXR) and chest computed tomography (CT), and pharyngeal and stool samples were tested for SARS-CoV-2 by RT-PCR. Both CXR and CT scans were normal, while surprisingly the results of both SARS-CoV-2 tests were positive. Thus, the patient was isolated. As she had no respiratory symptoms, according to our national guideline [5], we decided to continue with conservative treatment.

On day 4 of admission, she had no vomiting and had good oral tolerance. Fever and bloody diarrhea had stopped. On day 7, SARS-CoV-2 tests for nasopharyngeal and stool samples were still positive. The patient was discharged after the parents were educated on how to continue respiratory and gastrointestinal isolation. She was tested every week for SARS-CoV-2 in nasopharyngeal and stool samples. Both test results were negative 32 days after the onset of symptoms.

## Discussion and conclusions

The novel coronavirus SARS-CoV-2 is a new member of the family *Coronaviridae*, genus *Betacoronavirus*, which was first described in a series of patients presenting with pneumonia in China. Similar to lessons learned from severe acute respiratory syndrome (SARS), although at a lower frequency, SARS-CoV-2 has tropism to the GI tract [3, 6]. The angiotensin-converting enzyme 2 (ACE2) receptor is believed to be responsible for the entrance of SARS-CoV-2 into the cells, and it is abundantly expressed in the GI tract epithelium. This may account for the GI symptoms of COVID-19 patients and the potential fecal–oral transmission of the disease [7, 8]. Indeed, there have been some pediatric cases with a confirmed diagnosis of COVID-19 who did not show respiratory symptoms as their initial and main presentations. They may initially be considered common pediatric illnesses other than COVID-19, such as febrile seizure or gastroenteritis, and lead to the spread of the disease in the community and among medical staff [9].

GI symptoms have been reported in children with COVID-19 either with or without respiratory symptoms. The most common GI symptoms were anorexia, vomiting, and diarrhea [2, 4, 10]. A few cases of GI bleeding have also been reported [2]. Although pediatric cases of COVID-19 with GI symptoms including abdominal pain and GI bleeding, and especially diarrhea, have been reported, the index case presented with dysentery with no clinical or radiological respiratory involvement, and to date, no similar case of dysentery as the only presentation in children with COVID-19 has been reported. Alternatively, different infectious etiologies might have caused dysentery and fever. However, no enteric bacterial pathogens or parasites other than SARS-CoV-2 were recovered from stool analysis and culture. The possibility of pseudomembranous colitis induced by the introduction of ceftriaxone was weak, as the patient had clinical and laboratory evidence of colitis prior to the administration of ceftriaxone.

Some physicians have attributed the GI symptoms to the hypoxia, as cell necrosis from tissue hypoxia may cause gastrointestinal mucosal cell damage, resulting in ulceration and hemorrhage [2]. However, our patient had no hypoxia and was not critically ill, and this may show that dysentery was the primary result of COVID-19 and that it can be considered as a new feature of this virus.

No respiratory symptoms were seen and the patient was not in critical condition; therefore, we did not use any antiviral treatment or hydroxychloroquine, as not only has the efficacy of these drugs on COVID-19 not yet been proven, but they may have significant adverse effects. We also followed the patient until both nasopharyngeal and stool tests were negative to determine the duration of viral shedding and the required isolation period. In many cases of COVID-19, viral shedding can continue beyond 14 days with positive RT-PCR tests; nevertheless, the follow-up of every patient for long periods of time in routine practice is not possible. On the other hand, the positive RT-PCR tests beyond the resolution of symptoms may not necessarily indicate the possibility of disease transmission, as in some cases of COVID-19, the high viral load at admission can account for this finding.

In conclusion, pediatric patients with COVID-19 usually do not present with typical clinical features of the infection, which is respiratory involvement. Dysentery may be the only presentation of this disease, and long-term isolation should be considered, as the viral shedding may last for more than a month. We aimed to increase awareness among pediatricians and other clinicians in considering COVID-19 as a possible etiology for abdominal pain and dysentery in children.

## Data Availability

The data sets used during the current study are available from the corresponding author on reasonable request.

## Abbreviations

ACE2: Angiotensin-converting enzyme 2
COVID-19: Coronavirus disease 2019
CRP: C-reactive protein
CT: Computed tomography
CXR: Chest X-ray
GI: Gastrointestinal
HPF: High-power field
RBC: Red blood cell
RT-PCR: Reverse transcriptase polymerase chain reaction
SARS-CoV-2: Severe acute respiratory syndrome coronavirus 2
WBC: White blood cell
